# Age- and sex-specific physiological cardiac remodeling: the search for the Fountain of Youth

**DOI:** 10.1007/s00392-024-02431-4

**Published:** 2024-03-18

**Authors:** Philipp Markwirth, Bernhard Haring

**Affiliations:** 1 Department of Medicine III, Saarland University Hospital, Homburg, Saarland Germany; 2 Department of Epidemiology and Population Health, Albert Einstein College of Medicine, Bronx, NY USA

## Abstract

Figure: Concepts of cardiac aging. Abbreviations: E/e’, transmitral early velocity to early diastolic mitral annular velocity; EF, ejection fraction; LV, left ventricular; LVEDD, left ventricular end-diastolic diameter; M/V left, ventricular mass to volume ratio.

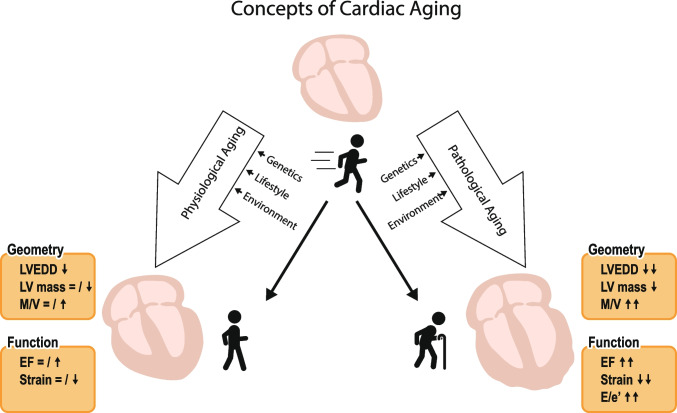

In healthy individuals without cardiovascular disease, aging is accompanied by a particular pattern of ‘physiological’ cardiac remodeling [1]. Prior cardiac magnetic resonance (CMR) data on 5004 participants from the MESA (Multi-Ethnic Study of Atherosclerosis) study found that with increasing age, left ventricular (LV) mass, stroke volume, and end-diastolic volumes decline despite a small increase in ejection fraction (EF) [1]. Interestingly, even though LV mass declined, LV mass to volume increased indicating higher LV stiffness. The underlying mechanisms and clinical implications of this remodeling remain elusive as systematic investigations are largely missing, and to this date, evidence was mostly stemming from US population–based cohorts. [1, 2]

In this issue of *Clinical Research in Cardiology*, Grassow et al. used a German cohort consisting of 140 healthy individuals to study the influence of sex and age on cardiac remodeling with CMR [3]. Individuals with known cardiovascular risk factors or established cardiovascular disease were excluded from the study. Similar to the findings from the MESA study [1], the authors report that with increasing age, LV end-diastolic volume indices as well as LV stroke volume indices decreased in both men and women, while LV mass remained largely unchanged. Again, LV mass-to-volume ratio was observed to increase over time, with men exhibiting a more prominent decline in LV stroke volume compared with women. Right ventricular (RV) end-diastolic volume indices decreased at a similar rate as LV volume indices in both sexes.

In contrast to physiological aging, “pathological” age-related changes in cardiac geometry and function are highly associated with the presence of traditional risk factors such as hypertension and diabetes and specific genetic conditions such as cardiac amyloidosis (Graphical abstract). Elevated LV afterload and uncontrolled hyperglycemia will eventually result in cardiac hypertrophy and cardiac fibrosis and path the way for adverse cardiac remodeling characterized by a decreased LV compliance over time. Subsequently, elevated LV filling pressures predispose to the development of heart failure with preserved ejection fraction (HFpEF) and pulmonary hypertension [2]. Affected individuals are at increased risk of cardiovascular death and predominantly suffer from dyspnea, increased fatigue, and exercise intolerance. [4, 5]

Although in the study by Grassow et al. as well as in other prior studies individuals with the presence of cardiovascular risk factors were excluded, it remains uncertain whether aging itself determines the specific pattern of “physiological” cardiac remodeling over time. In industrialized societies, systolic blood pressure increases with age, and this age-related rise in systolic blood pressure is commonly considered part of “normal” aging societies [6, 7]. However, it is much less pronounced in indigenous societies, suggesting that residual lifestyle and environmental factors may contribute more than widely recognized [8]. A prospective observational study in Tsimane forager-farmers, an indigenous tribe in Bolivia, found that in the absence of an industrialized environment, hypertension is far less prevalent compared to, e.g., US populations. This difference is attributed to a healthy lifestyle characterized by a great amount of physical activity, a predominantly plant-based diet low in sodium, and the absence of overweight or obesity. Acculturation to a Western lifestyle has been shown to result in a steeper relationship between age and systolic blood pressure [9]. Hence, “physiological” age-related cardiac changes likely reflect the cumulative exposure to risk factors during a person’s course of life and probably greatly vary between societies and cultures.

To this date, little is known about age-related sex-specific cardiac changes [10]. Female hearts display smaller chamber sizes, increased left LV wall thickness, and greater diastolic dysfunction [10]. Women are consequently more susceptible to developing HFpEF as compared to males who tend to develop heart failure with reduced ejection fraction (HFrEF) [10–13]. The reasons for this difference are likely explained by the lifetime course of sex hormones as women after menopause show an abrupt increase in blood pressure (eventually exceeding that of men) [6, 9]. Estrogen has been shown to reduce collagen synthesis in vitro in murine and human cardiac fibroblasts, suggesting that a drop in estrogen levels after menopause may be responsible for increased LV interstitial collagen deposition. [14]

Despite its limitations, the study by Grassow et al. provides us with valuable data on age- and sex-related structural and functional cardiac changes using a middle-European cohort. Clinicians should be aware of these sex- and age-specific differences. Reference values for cardiac geometry and function at older age can be derived which are relevant for daily decision-making on whether certain findings are to be considered physiological or already pathological.
